# Stress & strain in mechanically nonuniform alveoli using clinical input variables: a simple conceptual model

**DOI:** 10.1186/s13054-024-04918-y

**Published:** 2024-04-29

**Authors:** John J. Marini, Patricia R. M. Rocco, Lauren T. Thornton, Philip S. Crooke

**Affiliations:** 1 Department of Pulmonary and Critical Care Medicine, University of Minnesota, Minneapolis, St Paul, MN USA; 2 Laboratory of Pulmonary Investigation, Carlos Chagas Filho Institute of Biophysics, Federal University of Rio de Janeiro, Rio de Janeiro, Brazil; 3 Department of Mathematics, Vanderbilt University, Nashville, TN USA

**Keywords:** Ventilator induced lung injury, VILI, Mechanical power, Mechanical lung stress, Mechanical lung strain, Non-homogeneity, Stress amplification, ARDS

## Abstract

Clinicians currently monitor pressure and volume at the airway opening, assuming that these observations relate closely to stresses and strains at the micro level. Indeed, this assumption forms the basis of current approaches to lung protective ventilation. Nonetheless, although the airway pressure applied under static conditions may be the same everywhere in healthy lungs, the stresses within a mechanically non-uniform ARDS lung are not. Estimating actual tissue stresses and strains that occur in a mechanically non-uniform environment must account for factors beyond the measurements from the ventilator circuit of airway pressures, tidal volume, and total mechanical power. A first conceptual step for the clinician to better define the VILI hazard requires consideration of lung unit tension, stress focusing, and intracycle power concentration. With reasonable approximations, better understanding of the value and limitations of presently used general guidelines for lung protection may eventually be developed from clinical inputs measured by the caregiver. The primary purpose of the present thought exercise is to extend our published model of a uniform, spherical lung unit to characterize the amplifications of stress (tension) and strain (area change) that occur under static conditions at interface boundaries between a sphere’s *surface* segments having differing compliances. Together with measurable ventilating power, these are incorporated into our perspective of VILI risk. This conceptual exercise brings to light how variables that are seldom considered by the clinician but are both recognizable and measurable might help gauge the hazard for VILI of applied pressure and power.

**Supplementary Information:**

The online version contains supplementary material available at 10.1186/s13054-024-04918-y.

## Background and main text

**Fig. 1:**
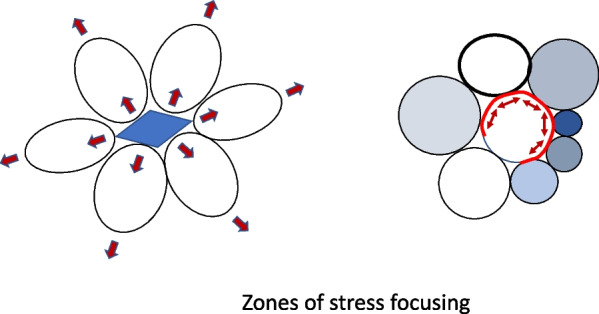
Stress interface concept. Left: An unyielding lung unit surrounded by normal ones inflated by the same airway pressure exert amplifying forces on both at their interface. Right: Multiple stress interfaces between a one flexible lung unit surrounded by open but less compliant ones of different dimensions and individual compliances (shaded). Such interfaces amplify the surface stresses at zones of contact

Clinicians currently monitor pressure and volume at the airway opening, assuming that these observations relate closely to stresses and strains at the micro level. Indeed, this assumption forms the basis of current approaches to lung protective ventilation [1]. Nonetheless, although the airway pressure applied under no-flow conditions may be the same everywhere in healthy lungs, the pressures and stresses within a mechanically non-uniform ARDS lung are not [2, 3]. Similarly, the potential for ‘power’ of ventilation to damage the lung has been acknowledged as highly relevant to the risk of ventilator induced lung injury (VILI), and its components of tidal volume, airway pressure, flow and cycling frequency are easily measured [4, 5]. As currently described for clinical purposes, however, ‘power’ is actually the cumulative energy delivered per minute by repeated tidal cycles that generate the mechanical forces needed to ventilate [5]. Therefore, to better assess ventilator-induced lung injury (VILI) hazard, the total ‘power’ monitored in the ventilator’s circuit needs to be considered in relation to the regional micro stresses (tensions at the alveolar boundary) and micro strains (resulting increments of area) that occur at the local level with each inflation cycle (intra-cycle power) [6]. Moreover, in theory, a refined index of ventilating hazard from measured power would be affected not only by the size of the ventilated compartment (‘baby lung’) [7] and its pressure threshold for injury [8, 9], but also by its proportion of interfaces where stress is focused and amplified between tissues having different receptivity to stretching (‘surface element compliances’) [10] (Fig. 1). A more clinically informative model of VILI risk would therefore include not only total power, as currently defined, but also estimates for the concentrated *specific* power (power applied to the baby lung [6, 11, 12], stress amplification at the alveolar level [3, 10] and the proportion of the ventilated baby lung experiencing such stress-augmented interfaces.

The term ‘mechanical stress’ describes the distribution of forces exerted in a solid or fluid body being deformed (‘strained’) as a result of external loads [13]. For ventilation, stress is analogous to pressure for forces *perpendicular* to the surface of contact (compressive, radial, or tensile stress) and strain is the resulting expansion (volume change) relative to the baseline condition. Forces *within* the plane of the surface where the cells and extracellular matrix reside may be considered ‘hoop stresses’ associated with changes of tension and area [14]. It follows that while pressure is force per unit of area, tension is force per unit of length.

**Fig. 2:**
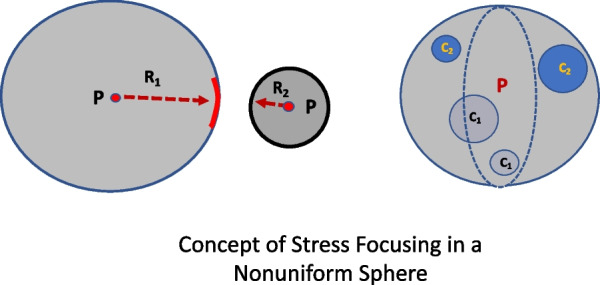
Mechanical Inhomogeneity Left: Two-dimensional representation of the relationship between the compliant (blue, thinner outline) and less compliant (red, thicker outline) surface elements that comprise the interface between them. R_1_ and R_2_ are the radii corresponding to hypothetically independent passive spheres distended by the same pressure, P. The ratio R_1_/R_2_ is the tension multiplier at the interface of their merged surface elements. Right: Higher compliance (lighter shading) and lower compliance (darker shading) surface elements of similar size. The interface amplification relationships summarized in Table 1 apply to each circular pairing. Note that an irregular surface element of any shape can theoretically be transformed into a circular element of the same area

In a simplified mathematical model using the clinically relatable variables of pressure and volume, we previously recognized those geometry-defined differences of force distribution to propose a conceptual shift from pressure to tension and from volume to area when considering the stress–strain changes of tissue energy at the alveolar periphery (the “shell”) [15]. While valid for a uniform alveolus modeled as a hollow, thin-walled sphere in which tension (T) is the product of pressure (P) and sphere radius R: (T = PR/2), tidal forces distribute stress and strain unevenly in diseases such as ARDS. Stresses in that setting are focused and amplified at the interface between different surface elements, such as those created by contiguous flexible and less flexible units. *The ratio of these interfacial tensions is a ratio of forces, which can be viewed as a numerical indicator of stress amplification, a potential contributor to risk for damage* (Fig. 2).

On the basis of volume differences observed in the histology of healthy lung, Jere Mead and colleagues accurately reported a 10:1 relationship between the dimensions (volumes) of an alveolus fully distended by 30 cmH_2_O and one that is completely collapsed [10]. According to their estimates, the corresponding amplification of the pressure stress at the boundary of open and atelectatic units at that extreme might be fourfold the measured pressure value. Although it is tempting to translate such modeling calculations directly to the clinical problem of approximating stress and strain imposed on the lung by mechanical ventilation, to our knowledge, such a mathematical representation of heterogeneous stresses using principles and variables familiar to clinicians is not currently available.

The primary purpose of the present thought exercise is to extend our uniform spherical model [15] to characterize the amplifications of stress (tension) and strain (area change) that occur at boundaries between a sphere’s *surface* elements that have differing compliances. As examples, these may be caused by local external features such as atelectasis, micro thrombosis, consolidation, edema, or fibrosis in other contiguous tissue adjacent to the spherical unit in question. In describing such a non-uniform spherical unit, we make several key assumptions: time invariant (static) conditions, open architecture not subject to tidal re-opening/closure, linear pressure–volume relationships, and unchanging shape morphology during expansion.

Finally, as another conceptual step toward better defining the VILI hazard under dynamic conditions, we also describe the relevant place of *specific* elastic power (as opposed to total power measured in the external circuit) in generating such damaging forces within the baby lung.

## Model using a mechanically ventilated nonuniform sphere to quantify focused stress & strain

To model the clinical hazard to a mechanically heterogeneous environment more realistically requires modification of the ‘uniform sphere’ model we previously derived [15] by applying ‘amplification multipliers’ to its expressions of tension (the product of pressure and sphere radius) and surface area change that characterize peripheral stress and strain, respectively. Mathematically, these amplification ‘multipliers’ are the multiplicative co-factors of measures of stress, strain, tension, and energy. This approach assumes that the discontinuous interface occurs where a less flexible region of reduced compliance ‘C_2_’ meets a region of similar baseline dimension within the surface (‘shell’) of a larger, more flexible sphere having compliance ‘C_1_’. The volume of the non-uniform sphere is designated V_1_. Both regions (surface elements) are exposed to the same applied pressure difference but because of their differing compliances experience different tensions (stress) and area expansions (strain) at their interface (Fig. 2). Theoretically, the less flexible, arc-like surface element would naturally form part of the shell of a smaller uniform sphere having volume V_2_ were that same pressure applied to it in isolation (Fig. 2). The interface may be an arc segment of any length and differing flexibility which is incorporated into the sphere’s ‘shell’ (fused at the intersection). In other words, the segment has a different incremental expansion response to gas pressure than the remainder of the shell but does not form part of a separate (smaller) sphere. It follows that the surface area of that less flexible surface element embedded in the non-uniform sphere would also expand less (and experience greater tension) than a corresponding area of the shell that surrounds it in response to the same applied pressure difference, ∆P. These assumptions ignore deformation of either spherical shape resulting from stretch above relaxed volume. Because tidal compliance is (∆V/∆P), ∆V_1_/∆V_2_ = C_1_/C_2_. Note that these dissimilar surface elements undergo different amounts of stretch in response to the pressure increment, generate shear stresses at their interface, and store different amounts of elastic energy in the same C_1_/C_2_ ratio that applies to volume.

Estimates of how those different elastic energies within the sphere are partitioned into tension (stress) and area (strain) requires estimates of their respective radii, R_1_ and R_2_. In a sphere of any dimension, volume equals 4/3 π R^3^ and area is 4 π R^2^. Consequently, if considered independently from one another at the same static pressure, both the local tensions and areas of the disparate surface elements can be derived from knowledge of the radii of their respective volumes and compliances (Fig. 2). As detailed in the on-line Additional file 1, the interface stress amplifier then would be estimated as (∆V_1_/∆V_2_)^1/3^ = (C_1_/C_2_)^1/3^ and the strain multiplier as (C_1_/C_2_)^2/3^. Importantly, if we concentrate on the *elastic energy* input to this non-uniform shell, ∆(P×V) = ∆(T×A), the *tension (stress)* multiplier (C_1_/C_2_)^1/3^ would be the co-factor of the *area (strain)* multiplier: (C_1_/C_2_)^2/3^. The product of the tension and area multipliers (stress and strain ratios) is the ratio of elastic energies stored in the flexible (E_1_) and less flexible (E_2_) regions of the shell interface: E_1_/E_2_ = [(C_1_/C_2_)^1/3^ × (C_1_/C_2_)^2/3^)] = C_1_/C_2_. Appropriately, this estimate agrees with ‘P×V determined’ stored energies relating to their compliance-defined volumes. The ratio C_1_/C_2_ (equivalent to V_1_/V_2_) is a key input to our model’s estimates of amplifiers of stress and strain. Therefore, as a first approximation, its possible range would span the tenfold volume range found histologically by Mead and colleagues [10]. *Our conceptual hypothesis can be stated: In a mechanically non-uniform sphere, these compliance-driven expressions are the multipliers that cause stress and strain to focus where different surface elements interface.*

### A multi-component hazard index of VILI & ‘damaging’ power

#### Energy and power component

To assess its actual VILI hazard, total power—currently defined for clinical purposes as the product of frequency and inflation energy per cycle—needs to be considered in the context of its relation to the micro stresses (tensions) and micro strains (area increments) occurring at the local level. For ARDS, such a VILI hazard would be affected by the relative size of the ventilated ‘baby’ lung, as its reduced aerating capacity concentrates the measured ventilating power. Such concentration may deliver damaging energy beyond the pressure threshold in the form of amplified surface stress and strain [7, 10, 12]. Therefore, viewed selectively from the standpoint of measurable *damaging energy* that generates intolerable stretch, the concerns are power, critical pressure threshold, and baby lung size. Conceptually, the relative size of the baby lung is reflected by C_obser_/C_pred_, where C_pred_ is the patient’s predicted compliance value when healthy [16, 17], and C_obser_ is the value actually observed during ventilation at ‘optimized’ PEEP [18]. A common convention is to assume that compliance (∆V/∆P) relates more closely to the number of open units than to stiffness of individual units. If so, the ‘relative risk factor’ of power concentration for a given patient’s lung is C_pred_/C_obser_. Although not commonly measured, the relative proportion of tidal volume compared to actual inspiratory capacity might yield a complementary and measurable estimate of the relative size of the baby lung.

#### Stress and strain component

**Fig. 3:**
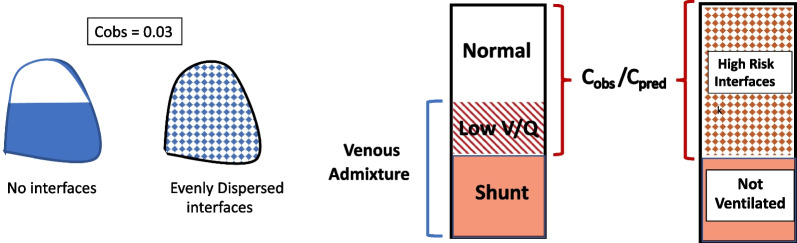
Two extremes of interface distribution. Left: Complete regional separation of normal from abnormal ventilation (minimal interfacing) versus complete dispersal (maximized interfacing) of ventilating abnormalities among the normally ventilating units of the baby lung (stippled). Right: Illustration of how gas exchange information from venous admixture comprised of true shunt and low V/Q units might help to estimate the proportion of high-risk interfaces within the baby lung. Together, the normal and abnormal units within the ventilated lung (C_obs_/C_pred_) comprise the indicated non-shunt fraction of total predicted lung volume

From the selective perspective of the individual lung unit where stress and strain that arise from intracycle elastic energy may cross the damaging threshold, however, the factors to consider are inflation pressure, tension, stress focusing, and prevalence of interfaces in the ventilated environment. Building an indicator of damaging local stress in stepwise fashion, starting from measured pressure would require estimates for (1) alveolar tension (as described in our prior ‘uniform sphere’ model [15]); (2) interfacial focusing/amplification; and (3) proportion of high-risk ventilated interfaces within the baby lung. For a given baby lung of any size, the proportion of interfaces that experience amplification might range from negligible in an ARDS lung in which an uninterrupted block of ventilated units with normal compliance is completely separated from those that are unventilated, to a fraction that is influenced by the number of refractory units evenly (diffusely) distributed among open ones (Fig. 3). The formulae modeling these stress and power elements of the multi-component hazard risk are summarized in Table 1. Note that an intervention might simultaneously influence one or more of these key hazard components in a direction that opposes the others. For example, raising PEEP might help by recruiting unstable units to increase their *number* in the ventilated baby lung even as PEEP adversely raises alveolar tension and amplifies interfacial stress (Examples are provided in the Additional file 1).

**Table 1:** Model Components and Associated Risk Amplifiers (see text). C_1_ = Compliance of the more flexible interfacial surface; C_2_ = Compliance of the opposing surface at the interface; C_obs_/C_pred_ = Ratio of observed to predicted (normal) compliance values; Ca_O2_ = Oxygen content of systemic arterial blood; Cv_O2_ = Oxygen content of mixed venous (systemic) blood; Cc_O2_ = Oxygen content of pulmonary capillary blood in a normally ventilated and perfused lung unit

Model component	Baseline variable	VILI hazard multiplier
Stress	Tension	(C_1_/C_2_)^1/3^
Strain	Area	(C_1_/C_2_)^2/3^
Surface energy	Tension × Area	C_1_/C_2_
Baby lung size	Compliance	C_obs_/C_pred_
Proportion of high-risk interfaces	Venous admixture	[(Cc_O2_–Ca_O2_)/(Cc_O2_–Cv_O2_)]

#### Proportion of high stress interfaces component

While acknowledging the difficulty of such an approximation, we suggest that standard gas exchange formulae that involve measurable variables might help determine the proportion of the aerated baby lung at greatest risk for the interfacial stress amplification discussed earlier (Fig. 3). The venous admixture that gives rise to hypoxemia is generated by both true shunt and open but inadequately ventilated (low V/Q units). Venous admixture is calculated as (Cc_O2_-Ca_O2_)/(Cc_O2_-Cv_O2_), where Cc_O2_ Ca_O2_ and Cv_O2_ are O_2_ contents of pulmonary capillary, systemic arterial and mixed central venous blood, respectively at a given fraction of inspired oxygen [19]. For our modeling purpose we hypothesize that the proportion of *non-aerated* units is the ‘true shunt’ fraction and thus exempt from inflation injury, as opposed to the fraction of low V/Q units at greater risk for interfacial stress amplification during tidal expansion. The ‘true shunt’ fraction is traditionally estimated at bedside by re-measuring venous admixture after administering pure inspired oxygen, thereby eliminating any hypoxic contribution from poorly ventilated units [19]. Theoretically, the proportion of the aerated baby lung that has interface exposure would then be: [(Cc_O2_-Ca_O2_)/(Cc_O2_-Cv_O2_) – (true shunt fraction)]. Alternatively, though less appealing but more simply at the bedside, one might assume all ventilated alveoli that comprise the baby lung are normally perfused, and all others (both shunt and low V/Q) are not. The latter are abnormal units and therefore points of mechanical heterogeneity that are scattered evenly and diffusely throughout the ventilated space. The proportion of stress focusing interfaces within the ventilated baby lung would then be simply: (Cc_O2_-Ca_O2_)/(Cc_O2_-Cv_O2_) [see Additional file 1 example].

### Limitations

This conceptual exercise brings to light how variables that are seldom considered by the clinician but are both recognizable and measurable might help gauge the hazard for VILI of applied pressure and power. To our knowledge this simplified, multi-part model is the first attempt to do so for the caregiver who manages the mechanically heterogeneous environment of injured lungs (ARDS). However, we understand and strongly emphasize that our assumptions and modeling are neither precise descriptors of micromechanics nor intended for immediate clinical use. Quite obviously, they have limited correspondence with the complex geometry that characterizes the actual biological environment of the injured lung. Our highly simplified approximations consider only static elastic forces, ignore dynamics and local differences of transpulmonary pressure, and depend on multiple assumptions. For example, thin-walled *spheres* are assumed in order to apply the LaPlace formula to the interface between surface elements [20]. However, neither the whole lung nor its constituent units are spheres exposed to a single transpulmonary pressure; biological lung unit contours are both irregular and interdependent, with variable topography of corners and interfaces. Moreover, while the transpulmonary pressures and relative compliances of each surface element of an interface are likely to lie within known ranges [10, 21], in actuality, these vary in accordance with their gravitational positions within the lung and immediate local environments. Estimating baby lung size indirectly from respiratory system compliance is clearly another approximation, as is calculating the proportion of the aerated baby lung with high-risk interfaces from gas exchange measurements. Importantly, the assumption of quasi-normal specific compliance of all aerated baby lung subunits [7], while perhaps reasonable in the first edematous stage of ARDS, may not apply in the later stages of organizing ARDS.

## Conclusion

**Fig. 4:**
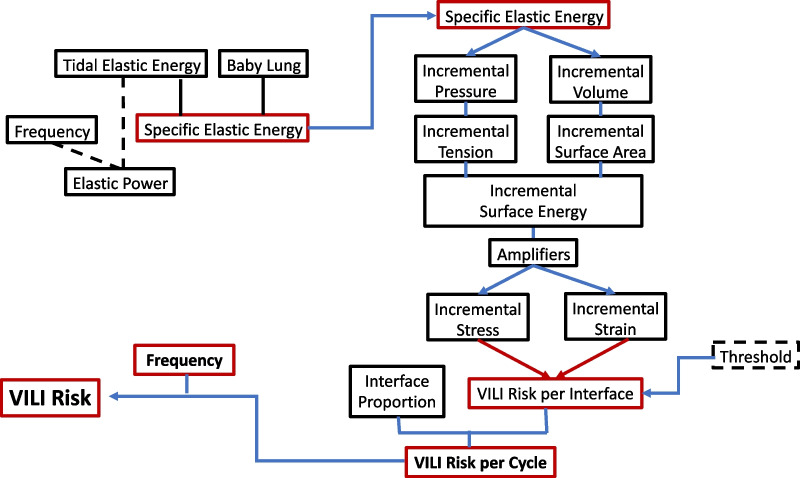
Relationships among the various determinants of risk for ventilator induced lung injury (VILI). VILI risk from mechanical forces relates directly to input elastic energy per inflation per cycle and frequency but inversely to baby lung size. The resulting incremental stresses and strains at the alveolar periphery cause damaging stretch during each cycle when amplified and threshold is exceeded [For explanation, see text]

In principle, estimating actual tissue stresses and strains that occur in a mechanically non-uniform environment should account for factors beyond the measurements from the ventilator circuit of airway pressures, tidal volume, and mechanical power (Fig. 4). A first step for the clinician requires consideration of lung unit tension, power concentration, and stress focusing. With reasonable approximations, better understanding of the value and limitations of presently used general guidelines for lung protection may eventually be developed for the individual patient from clinical inputs recognized and measured by the bedside caregiver. Although only a conceptual first step, such modeling may help understand what eventually might constitute a true ‘lung protective’ approach to ventilation.

### Supplementary Information

**Additional file 1: **Derivation of Amplifiers.

## Data Availability

Not applicable.

## Abbreviations

A: Area
C: Tidal lung unit compliance
C_obs_: Observed compliance
C_pred_: Predicted (normal) compliance
C_1_: Compliance of the more flexible interfacial surface
C_2_: Compliance of the opposing less flexible interfacial surface
Ca_O2_: Oxygen content of systemic arterial blood
Cv_O2_: Oxygen content of mixed venous (systemic) blood
Cc_O2_: Oxygen content of capillary blood in normally ventilated and perfused lung units
P: Trans-pulmonary pressure
R: Sphere radius
T: Tension of an alveolar surface
V: Lung unit volume
V/Q: Ventilation to perfusion ratio
