# Supplementary material for: Stress & strain in mechanically nonuniform alveoli using clinical input variables: a simple conceptual model
Source: Crit Care. 2024 Apr 29;28:141. doi: 10.1186/s13054-024-04918-y (PMC11057067; doi:10.1186/s13054-024-04918-y)
Supplement: Supplementary file 1 — Additional file 1: Derivation of Amplifiers. [file 13054_2024_4918_MOESM1_ESM.docx]

**Supplement**

**Stress and Strain Amplifiers of the Surface Element Interface: Derivation & Examples**

**Multipliers of Stress and Strain at the Interface**

The ∆ (distending Pressure (P) x Volume (V)) product relates to inflation energy, which at the surface is distributed as stress, (∆ tension (T)), and strain (∆ area (A)). The geometrical components of a sphere that relate to *volume and surface area* equal 4/3 π R^3^ and 4 π R^2^, respectively, where R is the sphere’s radius. Determining ‘R’, therefore, is central to both components of surface energy. At a given distending pressure (P), tension (T) at the surface of a hollow sphere is: T=PR/2.

Consider two different spheres inflated by the same pressure (P) that have two different volumes V_1_ and V_2_ with radii of ‘R_1_’ and ‘R_2_’, respectively [Figure]. Those radii are determined by their individual resting volumes and compliances (C_1_ and C_2_). When starting from a baseline of zero resting volume, it follows that their radii are needed to calculate the tensions (PR) and areas of these hypothetically independent spheres. Assign the more flexible larger sphere the volume V_1_ =PC_1_ and the less flexible, smaller volume sphere V_2_=PC_2_. Because

V=4/3 π R^3^, R_1_=[(3V_1_/4π)]^1/3^and R_2_=[(3V_2_/4 π)]^1/3^. Given that V=PC, these expressions can be rewritten: R_1_=[(3 PC_1_/4 π)]^1/3^and R_2_=[(3 PC_2_/4 π)]^1/3^

Simplifying by division to eliminate their common elements, the ratio of R_1_/R_2_ = (C_1_/C_2_)^1/3^ Recalling that stress is the ‘shell’ tension, T=PR/2, their surface elements would have different tensions in that same ratio. Consequently, **the tension multiplier (stress multiplier) at the interface, T_1_/T_2_ , would equal R_1_/R_2_ = (C_1_/C_2_)^1/3^.**

By the same reasoning and algebraic process, the *strain* multiplier that relates to area can be derived from the radii of the same two spheres, as Area = 4 π R^2^. Again, starting from a baseline of zero volume and pressure, this ratio is derived from their respective volumes at the same pressure, as governed by their input compliances: R_1_/R_2_ = (∆V_1_/∆V_2_) ^1/3^ = (C_1_/C_2_)^1/3^. R_1_= [(3 PC_1_/4 π)]^1/3^and [R_2_=[(3 PC_1_/4 π)]^1/3^. Therefore, the areas are Area_1_= 4 π R_1_^2^ = 4 π [(3 PC_1_/4 π)] ^2/3^ Area_2_= 4 π R_2_^2^=4 π [(3 PC_1_/4 π)]^2/3^. After simplification, **the corresponding area ratio (strain multiplier) at the interface is: (C_1_/C_2_)^2/3^ The product of the stress and strain ratios is the ratio of their inflation elastic energies [(C_1_/C_2_)^1/3^ ] x [(C_1_/C_2_)^2/3^] = (C_1_/C_2_).** Note that the ratio of elastic energy for these distinct surface elements is determined by the corresponding PV products of the two hypothetical sphere volumes, PV_1_ and PV_2_, which again is (C_1_/C_2_). Because radial and surface energies are identical, this equivalence is expected.

Estimating the numerical ranges of possible interface multipliers for stress and strain

For flexible and atelectatic units at the interface of a healthy lung region, these multipliers are functions of the Mead-approximated *volume ratio*, which ranged from 1 to a maximum of 10 (ref). In other words, in a given horizontal plane within the lung, the range of possible tension (stress) ‘multipliers’ would be the cube root of 10 (2.15) and the cube root of 1 (1.00). By similar reasoning, the (strain) multiplier at the interface that relates to *area* (4 π R^2^) is (C_1_/C_2_)^2/3^ and ranges between 10^2/3^ (4.64) and 1.00^2/3^ (1.00).

**Examples of VILI Hazard Calculation**s

**1) Moderate** **ARDS** : V_T_ = 0.430 L; f =20/min; V_E_ =8.6 L/min; PEEP 7.5 cmH_2_O; C_obser_ 0.045 L/cmH_2_O; C_pred_ 0.10 L/cmH_2_O; C_1_/C_2_= 0.090/0.045; Alveolar (plateau) pressure: 17 cmH_2_O; FiO_2_ 0.70; SaO_2_ 92%

| **VILI Hazard Component** | **Baseline Variable** | **Formula** | **Value** | **Energy** |
| --- | --- | --- | --- | --- |
| Interface Stress Multiplier | Tension | (C_1_/C_2_)^1/3^ | 1.26  **Stress & Strain** |  |
| Interface Strain Multiplier | Area | (C_1_/C_2_)^2/3^ | 1.59 |  |
| Surface Energy Multiplier | Tension x Area | C_1_/C_2_ | 2.0 |  |
| Proportion of High-Risk Interfaces | Venous Admixture | [(Cc_O2_ - Ca_O2_)/(Cc_O2_ - Cv_O2_)] | 0.33 |  |
| Relative Baby Lung Size | Compliance | C_obs_/C_pred_ | 0.45 |  |
| Power | Joules/min | V_E_ × (*V*_T_/2C_obs_ + PEEP) | 10.6 |  |
| Specific Power | Joules/min | V_E_ × (*V*_T_/2C_obs_+ PEEP) / (C_obs_/C_pred_) | 21.2 |  |

2) **Moderate ARDS**: V_T_ = 0.430 L; f =25/min; V_E_ =10.8 L/min; PEEP = 12 cmH_2_O; C_obser_ 0.054 L/cmH_2_O; C_pred_ 0.10 L/cmH_2_O; C_1_/C_2_= 0.090/0.054; Alveolar (plateau) pressure: 20 cmH_2_O; FiO_2_ 0.70; SaO_2_ 95%

| **VILI Hazard Component** | **Baseline Variable** | **Formula** | **Value** |
| --- | --- | --- | --- |
| Interface Stress Multiplier | Tension | (C_1_/C_2_)^1/3^ | 1.18  **Stress & Strain** |
| Interface Strain Multiplier | Area | (C_1_/C_2_)^2/3^ | 1.41 |
| Surface Energy Multiplier | Tension x Area | C_1_/C_2_ | 1.67 |
| Proportion of High-Risk Interfaces | Venous Admixture | [(Cc_O2_ - Ca_O2_)/(Cc_O2_ - Cv_O2_)] | 0.20 |
| Relative Baby Lung Size | Compliance | C_obs_/C_pred_ | 0.54  **Energy** |
| Power | Joules/min | V_E_ × (*V*_T_/2C_obs_ + PEEP) | 17.2 |
| Specific Power | Joules/min | V_E_ × (*V*_T_/2C_obs_+ PEEP) / (C_obs_/C_pred_) | 31.9 |

3) **Severe ARDS**: V_T_ = 0.336 L; f = 30/min V_E_ = 10.1 L/min; PEEP=18 cmH_2_O; C_obser_ 0.028 L/cmH_2_O;

C_pred_ 0.10L/cmH_2_O; C_1_/C_2_= 0.080/0.020; Alveolar (plateau) pressure: 30 cmH_2_O; FiO_2_ 0.80; SaO_2_ 90%

| **VILI Hazard Component** | **Baseline Variable** | **Formula** | **Value** |
| --- | --- | --- | --- |
| Interface Stress Multiplier | Tension | (C_1_/C_2_)^1/3^ | 1.58  **Stress & Strain** |
| Interface Strain Multiplier | Area | (C_1_/C_2_)^2/3^ | 2.53 |
| Surface Energy Multiplier | Tension x Area | C_1_/C_2_ | 3.99 |
| Proportion of High-Risk Interfaces | Venous Admixture | [(Cc_O2_ - Ca_O2_)/(Cc_O2_ - Cv_O2_)] | 0.40 |
| Relative Baby Lung Size | Compliance | C_obs_/C_pred_ | 0.28  **Energy** |
| Power | Joules/min | V_E_ × (*V*_T_/2C_obs_ + PEEP) | 24.2 |
| Specific Power | Joules/min | V_E_ × (*V*_T_/2C_obs_+ PEEP) / (C_obs_/C_pred_) | 86.6 |

4) **Very Severe ARDS**: V_T_ = 0.270 L; f = 35/min; V_E_ = 9.45 L; PEEP 20 cmH_2_O; C_obser_ 0.018 L/cmH_2_O;

C_pred_ 0.100 L/cmH_2_O; C_1_/C_2_ = 0.075/0.020; Alveolar plateau pressure: 35 cmH_2_O; FiO_2_ 0.80; SaO_2_ 90%

| **VILI Hazard Component** | **Baseline Variable** | **Formula** | **Value** |
| --- | --- | --- | --- |
| Interface Stress Multiplier | Tension | (C_1_/C_2_)^1/3^ | 1.55  **Stress & Strain** |
| Interface Strain Multiplier | Area | (C_1_/C_2_)^2/3^ | 2.42 |
| Surface Energy Multiplier | Tension x Area | C_1_/C_2_ | 3.75 |
| Proportion of High-Risk Interfaces | Venous Admixture | [(Cc_O2_ - Ca_O2_)/(Cc_O2_ - Cv_O2_)] | 0.40 |
| Relative Baby Lung Size | Compliance | C_obs_/C_pred_ | 0.18  **Energy** |
| Power | Joules/min | V_E_ × (V_T_/2C_obs_ + PEEP) | 26 |
| Specific Power | Joules/min | V_E_ × (V_T_/2C_obs_+ PEEP) / (C_obs_/C_pred_) | 144 |

V_T_ = tidal volume; f = frequency; V_E_ = minute ventilation; PEEP= positive end-expiratory pressure; C_obser_ = observed compliance; C_pred_ = predicted compliance; C_1_/C_2_ = ratio of compliances for surface elements higher and lower compliance; FiO_2_ =fraction of inspired oxygen; SaO_2_ = oxygen saturation of systemic arterial blood

**Reference**: Mead J, Takishima T, Leith D. Stress distribution in lungs: a model of pulmonary elasticity. J Appl Physiol 1970; 28: 596-608
